# Editorial: Healthy healthcare: opportunities and pitfalls of designing and conducting research and practice in healthcare settings?

**DOI:** 10.3389/fpsyg.2024.1514074

**Published:** 2024-11-22

**Authors:** Annet H. de Lange, Lise Tevik Løvseth, Marit Christensen, Asta Medisauskaite, Kevin Rui-Han Teoh

**Affiliations:** ^1^Berenschot, Utrecht, Netherlands; ^2^Department of Work and Organizational Psychology, Open University, Heerlen, Netherlands; ^3^Department of Psychology, Universidade da Coruña, A Coruña, Spain; ^4^Department of Psychology, Norwegian University of Science and Technology (NTNU), Trondheim, Norway; ^5^Hotel School of Management, University of Stavanger, Stavanger, Norway; ^6^Clinic of Mental Health, St. Olavs University Hospital of Trondheim, Trondheim, Norway; ^7^UCL Medical School, University College London, London, United Kingdom; ^8^Birkbeck Business School, Birkbeck, University of London, London, United Kingdom

**Keywords:** *Healthy Healthcare* research, multi-stakeholder, systemic approaches, multi-level, review

## Introduction

*Healthy Healthcare* advocates for the design of healthcare systems that are managed and financed in a way that is aligned with the available resources in order to improve both healthcare workers' health and the quality of patient care delivered (Løvseth and de Lange, 2020). Ultimately a balanced-based perspective taking into account patients, staff and the complex organization of the healthcare system should lead to a more resource-efficient delivery of high-quality healthcare services (Løvseth and de Lange, 2020; Løvseth and Teoh, 2023). This is imperative as healthcare systems globally are experiencing multiple challenges that compound each other, including poor staff retention and health, an aging population, more complex healthcare needs, the aftermath of the COVID-19 pandemic, and limited financial resources (Poon et al., 2022; European Commission, 2021).

The systems perspective advocated by *Healthy Healthcare* draws on the need to integrate three pillars within research and practice: (i) the healthcare system and its organization; (ii) healthcare workers' health and wellbeing; and (iii) the quality of care provided (Løvseth and de Lange, 2020; Løvseth and Teoh, 2023). To advance the work in this area, de Lange et al. (2020a) outlined seven areas of development. At its core, the primary call was (1) to move beyond examining only one (or even two) of the three pillars and instead recognize the existence of all three pillars by developing studies based on a system perspective. In doing so, there is (2) a need to incorporate designs that acknowledge factors and interventions across multiple levels of the organization. This means (3) situating leadership within *Healthy Healthcare;* (4) acknowledging the need for primary interventions; as well as (5) capturing the diversity within the healthcare workforce, (6) wellbeing measures, and the (7) methodologies and paradigms employed.

With this in mind, the current Research Topic aims to summarize the recent methodology and content related to *Healthy Healthcare* from an occupational health psychology research perspective. This offers a space for reflection on the action raised by de Lange et al. (2020a, 2024), and to consider the opportunities and pitfalls presented in some more contemporary research.

### Current issue

We start by describing the accepted articles before highlighting how the main findings relate to identified research gaps in methodology and content from the perspective of occupational health psychology research. As a result, we synthesize new evidence-based occupational health psychology practices and research that link the three important pillars of healthcare.

The first accepted article by Albrithen and Yalli focuses on the recognition of social work in health care. This paper covers two elements that were identified for future research initiatives (cf. de Lange et al., 2020a, 2024), namely: *Multilevel study design* by capturing the organizational resources and structural issues and how these aspects impact employees (i.e., their commitment), and *Capturing the diversity of healthcare workforce* by considering social care workers and being set in the Middle East. Using self-administered questionnaires, this study examined the relationships between two organizational-contextual aspects (i.e., organizational structural and organizational resources) among social workers in health care in Saudi Arabia. The authorspoint to the importance of organizational structural obstacles in sustaining a balanced and high quality care, including: (i) role/job design problems (e.g., lack of clear job guidelines and increased involvement with less professional work); (ii) rule/work-regulations structure in the workplace (e.g., inflexible work-related politics, rigid bureaucracy); and (iii) hospital power structures (e.g., outmoded and centralized hierarchy). These affect care by influencing practitioners' professional autonomy, the amount of client work they do, their linkages with other work units, and limit their participation in decisions related to their services and the work of the organization. Moreover, constraints of organizational resources were related to the lack of funding for services, inadequate staffing levels in the social work department, insufficient space and necessary work related equipment and means, and lack of support services in the workplace for psychosocial care; all of which were perceived by respondents to create difficulties for social work staff to practice their roles in an efficient manner (e.g., increased workload). The results highlighted associations between participants' opinions concerning organizational structure and resources, and respondents' feelings of commitment to their organization. In this particular study, we learn that organizational barriers are important elements to overcome in balancing staff and patient demands within healthcare. Although the study does not directly measure the three pillars of healthcare, it pinpoints relevant challenges that can be used to develop solutions or interventions for creating healthier healthcare.

The second accepted paper by Brulin et al., aimed to investigate in which way performance-based reimbursement (PBR) systems in Swedish healthcare services subjectively impacted (i) physicians' work, (ii) the occurrence of stress-induced exhaustion disorders among physicians, and (iii) patient care. In this context, the developments discussed in de Lange et al.' (2020a) work focus on two key areas: *advancing research* and creating new overarching theories based *on the system perspective of Healthy Healthcare, which incorporates the three pillars—system, employee, and patient care*; and *adopting a multilevel study design* that takes into account both financial systems and employee factors, such as performance and wellbeing. This mixed-method study collected data from a representative sample of Swedish physicians. Thematic analysis on an open-ended question about participants' PBR experience resulted in four themes: (i) money talks (i.e., healthcare is governed by money rather than patients' needs); (ii) patients are affected (i.e., care provision being steered toward less complicated and more healthy patients); (iii) medical morals are challenged; and (iv) PBR increase the quantity of illegitimate tasks. Logistic regressions showed that physicians for whom PBR had an impact on their work had a 2-fold higher risk of stress-induced exhaustion disorder. The findings of this study suggest that the current reimbursement systems in Sweden negatively influence not only physicians' work and health but also patients. This study not only addresses the organizational structural demands, but extends this to examine how the broader financial reimbursement system influences quality of healthcare within practice.

The third paper by Lundmark et al. related to three research initiatives outlined in de Lange et al. (2020a) work: *Situating leadership within Healthy Healthcare* by incorporating line manager roles in the intervention; *Primary-interventions* by looking at organizational level interventions to improve work environment and using participatory design to develop and improve it; and *Embrace different research methodologies and paradigms* by trialing pilot studies and using the findings to improve the intervention. The basis of the paper is the need to identify measures to mitigate these challenges faced within the healthcare sector, and in particular, the implementation of sustainable tools and processes to improve the work environment of healthcare organizations. In this paper, the authors present a feasibility study protocol for a joint training with pairs of healthcare line managers and their associated health and safety representatives in a Swedish healthcare organization. The objective of the training is to aid and advance the implementation of interventions to improve the work environment at the unit level. Following recommendations in the literature, the training is based on a stepwise approach that considers the specific context and focuses on the involvement of employees in creating interventions based on their needs. A central component of the training is the development of the pairs' collaboration in initiating and facilitating the interventions, drawing on an on-the-job train-the-trainer approach where participants go through the steps of a participatory intervention process over four workshops. Between these workshops, the pairs follow the same progressive steps together with their employees to develop and implement interventions at their unit. The overall aim of the pilot was to appraise its feasibility and to adjust the training before a potential scale-up. It showed that the preparation phase in creating healthy healthcare in terms of having a clear and joint stakeholder strategy is important in communicating and training for implementing relevant interventions in healthcare.

The fourth paper from Cooper et al. covers multiple elements that were also highlighted by de Lange et al. (2024), namely: *Multilevel study designs* by recognizing organizational factors and processes that can harm staff, *Capturing the diversity of the healthcare workforce* by involving wide range of staff, including HR and trade union representatives, *Situating leadership within Healthy Healthcare* by investigating management role in cultural change, *Primary-interventions* by testing organization level intervention, and *Embrace different research methodologies and paradigms* by bringing in organizational data and financial analysis to create an economic argument. This paper is an example of a *Healthy Healthcare* project that considers both the employee and organizational level in terms of perspectives and data collected. More specifically, this paper examines an intervention in a Welsh healthcare organization to reduce the number of employee investigations by taking a “last resort” approach. This stems from the concept of “avoidable employee harm,” which recognizes that poorly designed organizational systems and processes have the potential to harm employees. A range of associated improvement initiatives were developed to support behavior change among those responsible for determining whether an employee investigation should be initiated. Over a 13-month period, organizational records showed an annual reduction of 71% in investigation cases post-intervention, resulting in an estimated 3,308 sickness days averted annually and total estimated annual savings of £738,133. Analysis of survey data from those who attended training workshops to support the programme indicated that participants showed an increased awareness of the employee investigation process post-workshop and an understanding of the concept of avoidable employee harm. The programme is congruent with the *Healthy Healthcare* concept, as the study illustrates how its practices and processes have a beneficial impact on staff and potentially on patients by ensuring safe staffing levels and continuity of care. This study highlights wider issues for consideration, including the: (i) the role of Human Resources; (ii) taking a multi-disciplinary approach; and (iii) culture and practice.

The fifth and final in the current issue is work of Chua et al. covers a qualitative study examining the factors affecting job performance amongst junior doctors working for public healthcare institutions in Singapore. Within these institutions, junior doctors experience challenges with maintaining a balance in job demands and resources, leading to strain. Exploring the lived experiences of these junior doctors is essential when reviewing workplace and organizational factors that contribute to stress on an individual level, providing valuable insights to address these challenges effectively. Semi-structured interviews were conducted with 20 junior doctors in Singapore, ranging from house officers to senior residents. Framework analysis was performed to identify themes deductively based on the Job Demands-Resources (JD-R) Model. These themes shed light on how work demands, resources and personal factors influence the job performance of junior doctors and job satisfaction. The study offers valuable insights into the specific issues disrupting the job demands and resource balance in Singapore Public Healthcare Institutions and their correlation with job performance. The study highlights, similar to the other included papers, the importance of including a *multi-level perspective* in healthcare research (in this case the physician vs. organizational and work-related levels and perspectives). Furthermore, by analyzing more in-depth qualitative interviews, the study was able to show relevant new ways on how to improve working conditions for junior doctors, fostering their growth and engagement within the public healthcare system. Finally, the study emphasizes the importance of *Capturing the diversity of healthcare*, where the study indicates the additional demands junior doctors face in Singapore, including working hours that exceed those in Europe and Australia, and working cultures that are less supportive and more hierarchical than elsewhere.

## Discussion

This Research Topic aimed to address the opportunities and pitfalls in designing and evaluating *Healthy Healthcare* projects, a critical area of research as the healthcare sector faces mounting pressures globally. These include workforce shortages, increasing patient demands, and the need for system-wide reform to improve both employee wellbeing and patient care. The studies included in this issue underscore the complexity of adopting a holistic approach that addresses the three pillars of *Healthy Healthcare, namely*: (i) the healthcare system and its organization; (ii) healthcare workers' health and wellbeing; and (iii) the quality of care provided. While each study contributed meaningfully to the field, they also revealed several gaps and challenges that need to be addressed for a more comprehensive understanding and implementation of *Healthy Healthcare* environments.

The included studies show that conducting field research using a multi-stakeholder perspective in healthcare presents significant practical and theoretical challenges. Specifically, the shift from one or two stakeholder groups (typically employees and patients) to a broader, system-based approach that includes organizational perspectives proves difficult to achieve (von Thiele Schwarz et al., 2021). While most studies have successfully incorporated employee and patient data, the integration of organizational-level data, which is essential for a comprehensive understanding of the interdependencies between these pillars, remains limited. This gap is a crucial finding because without a systemic and multi-level stakeholder approach, interventions may fail to address the complex and interconnected nature of healthcare work environments (de Lange et al., 2024). Future research must focus on overcoming these barriers by developing more sophisticated methodologies that can capture the perspectives of all relevant stakeholders and apply a theoretical framework that supports this integrative approach (Løvseth and de Lange, 2020).

For instance, the study by Albrithen and Yalli on integrating social work in healthcare emphasizes the organizational barriers, such as unclear job roles and outdated power hierarchies, that hinder effective healthcare delivery. These structural issues are not unique to social work but are emblematic of the broader organizational challenges in healthcare systems that affect all stakeholders. This study highlights a critical pitfall: without addressing these systemic issues, efforts to improve healthcare work environments are likely to be undermined as they will not fully account for the organizational dynamics that impact both employees and patient outcomes (Nielsen and Miraglia, 2017; von Thiele Schwarz et al., 2021). However, the study also presents an opportunity by identifying areas where changes to organizational structure and resources can lead to healthier healthcare environments, thus reinforcing the need for system-wide interventions.

Similarly, the study by Brulin et al. on performance-based reimbursement systems in Swedish healthcare brings to light the unintended consequences of financial incentives. While PBR systems may improve organizational efficiency, they often do so at the expense of employee wellbeing and patient care quality. This aligns with the three-pillar approach by illustrating how organizational policies that prioritize financial outcomes can negatively impact both employees and patients (Neal et al., 2023). The study highlights a significant opportunity: redesigning incentive structures to promote a more balanced approach that considers employee health and patient outcomes alongside organizational goals (Løvseth and Teoh, 2023). Future research could build on these findings by exploring alternative reimbursement models that align with the principles of *Healthy Healthcare*.

Lundmark et al.'s feasibility study on leadership training within healthcare organizations provides another valuable perspective by demonstrating the effectiveness of participatory approaches in creating healthier work environments. By involving both healthcare managers and health and safety representatives in intervention design and implementation, this study offers a practical example of a multi-level approach that engages multiple stakeholders (von Thiele Schwarz et al., 2021). This participatory method not only improves organizational processes but also empowers employees, creating a more sustainable and healthy work environment (Christensen et al., 2019; de Lange et al., 2020b). This approach holds significant promise for future *Healthy Healthcare* interventions, as it offers a replicable model for engaging stakeholders at different levels of the organization in the co-creation of solutions.

The intervention by Cooper et al. to reduce employee investigations in Welsh healthcare by adopting a “last resort” approach highlights another key pitfall: poorly designed organizational processes that negatively affect both staff wellbeing and patient care. The significant reduction in employee investigations and the resulting improvements in staff wellbeing demonstrate the impact that targeted organizational changes can have on the overall health of the healthcare environment (Neal et al., 2023; Nielsen and Christensen, 2021). This study provides an opportunity for future research to explore similar interventions across different healthcare settings, particularly in terms of how organizational processes can be redesigned to support employee wellbeing and, by extension, patient care.

The qualitative study by Chua et al. on the job demands and resources of junior doctors in Singapore offers critical insights into how work demands and available resources affect job satisfaction and performance. By using the JD-R model, this study highlights the importance of addressing work demands and resources at both individual and organizational levels (Teoh et al., 2022). This approach offers a clear opportunity to develop interventions that target both personal and organizational resources, thereby improving the overall health of the healthcare work environment (de Lange et al., 2020b). However, the study also illustrates a common pitfall: the difficulty of simultaneously addressing the three pillars in a cohesive manner (Løvseth and Teoh, 2023; Teoh et al., 2023a). Most studies tend to focus on one or two pillars, which limits the potential impact of interventions. To overcome this, future research must develop more integrative approaches that consider the interplay between individual, organizational, and patient-level factors.

The recurring challenge across the studies is the difficulty in integrating all three pillars—employee, patient, and system—into healthcare research and interventions. This issue is compounded by the practical difficulties of data collection and the involvement of multiple stakeholder groups (de Lange et al., 2020a,b). While some progress has been made, particularly in terms of recognizing the importance of multi-stakeholder perspectives (de Lange et al., 2024), most research still tends to focus on one or two pillars, which limits the ability to fully understand and address the complexities of healthcare environments. This gap highlights the need for further theoretical development and the creation of practical strategies that can facilitate the incorporation of a wider range of stakeholders into healthcare research.

As noted by previous research, sustainable interventions that target the wider working environment tend to have a more lasting impact (Nielsen et al., 2017). However, the skills and resources needed to apply a systems perspective are still underdeveloped in much of the healthcare research community (Teoh et al., 2023b; Løvseth and de Lange, 2020). This is likely due to the interdisciplinary nature of such research, which requires collaboration across multiple fields, as well as the complexity of conducting interventions that span all three pillars. Identifying and learning from existing projects that embody the principles of *Healthy Healthcare*, as suggested by de Lange et al. (2024), is crucial for advancing the field and developing more effective interventions.

## Conclusion

In conclusion, this special issue reveals the growing interest in creating healthier healthcare environments and highlights several meaningful opportunities for further development. The included studies offer valuable insights into the challenges and opportunities associated with designing and implementing *Healthy Healthcare* interventions. However, they also underscore the need for more sophisticated approaches that fully integrate employee, patient, and organizational perspectives in future research. We hope that these articles will inspire further research and development in this critical area, ultimately leading to more effective and sustainable Healthy Healthcare systems.
